# Lysyl Oxidase Gene G473A Polymorphism and Cigarette Smoking in Association with a High Risk of Lung and Colorectal Cancers in a North Chinese Population

**DOI:** 10.3390/ijerph13070635

**Published:** 2016-06-28

**Authors:** Guoli Wang, Yanqing Shen, Guang Cheng, Haimei Bo, Jia Lin, Maogen Zheng, Jianmin Li, Yinzhi Zhao, Wande Li

**Affiliations:** 1 The Collage of Public Health, North China University of Science and Technology, Tangshan 063000, China; hbmywgl@163.com (G.W.); A1070320839@163.com (Y.S.); 2 The Clinic Medical College, North China University of Science and Technology, Tangshan 063000, China; Bohaimei@sina.com (H.B.); Zmg111222@sina.com (M.Z.); lijianmints@sina.com (J.L.); 3 The College of Life Science, North China University of Science and Technology, Tangshan 063000, China; linjia825@126.com; 4 Department of Biochemistry, Boston University School of Medicine, Boston, MA 02118, USA; yinzhi@bu.edu (Y.Z.); wandeli@bu.edu (W.L.)

**Keywords:** lysyl oxidase (LOX), lung cancer, colorectal cancer, colon cancer, rectal cancer, single nucleotide polymorphism (SNP), cigarette smoking

## Abstract

The relationship among the lysyl oxidase (LOX) G473A single nucleotide polymorphism (SNP), cigarette smoking and lung, colorectal, colon and rectum cancer susceptibility was studied in 200 cases of lung cancer, 335 cases of colorectal cancer including 130 cases of colon cancer and 205 cases of rectum cancer, and 335 healthy people in Tangshan, China. Peripheral blood DNA samples were collected, DNA sequencing and polymerase chain reaction-restriction fragment length polymorphism (PCR-RFLP) performed, followed by multivariate logistic regression analysis. In comparison to LOX473GG genotype carriers, individuals with LOX473AA exhibited a higher susceptibility to lung, colon-rectum, colon, and rectum cancers with OR values amounting to 3.84-, 2.74-, 2.75-, and 2.74-fold of the control, respectively. In the LOX 473AA-positive population, females were more susceptible than males to carcinogenesis with OR values (female vs. male): 5.25 vs. 3.23, 2.29 vs. 1.51, 2.27 vs. 1.45, and 2.25 vs. 1.53, respectively, for lung, colon-rectum combined, colon, and rectum cancers. LOX G473A polymorphism apparently elevated human sensitivity to cigarette smoking carcinogens for eliciting cancers in the lung and colon only. Thus, LOX G473A polymorphism positively correlates with carcinogenesis and it may be used as an ideal intrinsic biomarker for prediction or diagnosis of carcinogenesis in humans.

## 1. Introduction

Lysyl oxidase (LOX) (E.C.1.4.3.13), a copper-dependent enzyme secreted by fibrogenic cells, initiates the crosslinking of collagen and elastin, stabilizing the extracellular matrix (ECM). Increased LOX activity is associated with fibrotic diseases such as lung and liver fibrosis and atherosclerosis [1], whereas decreased LOX activity is associated with lathyritic agent-induced emphysema in animal models and with disorders of copper metabolism like Menkes syndrome. Thus, LOX plays a central role in organ morphogenesis and tissue repair [1]. Recently, LOX has been shown to display a dual function in carcinogenesis. As a tumor suppressor it inhibited Ha-*ras*-induced tumorigenesis [2] while as a tumor enhancer it facilitated tumor metastasis under hypoxia conditions [3]. LOX can oxidize lysine residues in various globular proteins other than collagen and elastin [1]. Oxidation of basic fibroblast growth factor (bFGF) by LOX blocked the proliferation of bFGF–stimulated cells and bFGF-autocrine transformed cells with a highly tumorigenic potential [4]. Purified mature bovine LOX displayed chemotactic activity for monocytes and vascular smooth muscle cells [5,6]. LOX existed within the nuclei, potentially targeting histone H1 and H2 and modulating the promoter activity of the collagen type III gene [7,8,9,10]. Thus, LOX acts as a critical intra- and extra-cellular effector carrying out multiple biological functions.

The single nucleotide polymorphism (SNP), i.e., a single-nucleotide substitution of one base for another, is the most common type of genetic variation among people. There are roughly 112 million SNPs falling within coding sequences or non-coding regions of genes in the human genome. Most SNPs may have no effect on health or development. However, some of these genetic differences, may be harmful to human life, for example, resulting in changes in protein amino acids (AA), and thus altering susceptibility to drugs, environmental factors, etc., increasing risk for development of particular diseases such as cancers [11]. The LOX gene locates at the chromosome 5q23.1–q23.2, containing 15,166 base pairs. There are approximately 643 SNPs found within the LOX gene. Several SNP sites are mapped in the LOX coding region, for example, C225G (C: cytosine, G: guanine), G409C, G473A (A: adenine), C476A, G816A, T924G (T: thymine), and A1135G, etc. Among these SNPs, G473A (rs1800449) displays the highest polymorphic frequency. It results in a change of Arg to Gln at the residue 158 in the LOX preproenzyme AA sequence (LOX Arg158Gln) [12,13]. The LOX G473A polymorphism has been reported to enhance human susceptibility to cancers or tumors in different organs and tissues such as lung, stomach, colon-rectum, breast, cervices, ovaries, and nervous and bone systems, etc. [14,15,16,17,18,19,20,21]. Furthermore, the human population with LOX G473A variants exhibited higher risk of suffering from some diseases such as coronary artery disease, keratoconus, retinal detachment, and vitreoretinopathy, etc. [22,23,24]. Therefore, the LOX G473A polymorphism as an intrinsic factor may play a critical role in carcinogenesis and pathogenesis for humans. 

Cancer is one of the major causes of death for humans. In 2012, there were 14 million new cases and 8.2 million cancer-related deaths worldwide. The amount of new cancer cases will predictably reach 22 million within the next two decades. More than 60 percent of the world’s new cancer cases occur in Africa, Asia, and Central and South America; 70 percent of the world’s cancer deaths also occur in these regions [25]. China has approximately 20% of the world’s population, but it has 22% of new cancer cases and 27% of the world’s cancer deaths. The Chinese National Cancer Registration Center reported an estimated 3.5 million newly diagnosed cases and 2.5 million deaths annually [26]. In the United States, there will be an estimated 1.65 million new cancer cases diagnosed and 0.59 million cancer deaths in 2015. Lung cancer is the most common cancer in China and the second most common cancer in the USA [25,26]. Colorectal cancer is the third most common cancer in the USA and the fifth most common cancer in China [25,26]. Cigarette smoking is the most common risk factor for lung cancer and also an important risk factor for colorectal cancer [27,28,29]. It is known that carcinogens activate serials of genetic and epigenetic mechanisms inducing transformation of normal cells to cancer cells. However, certain intracellular events and their underlying mechanisms for carcinogenesis remain unclear and should be understood. In light of LOX’s multiple biological functions and its dual role in carcinogenesis, we have investigated the frequency of LOX gene G473A polymorphism in the population of Tangshan, a city in northern China and identified the relationship between the LOX gene G473A polymorphism, smoking, and susceptibility to lung and colorectal cancers, two most common cancers in the world. Results indicated that the LOX G473A SNP increases the risk of lung, and colorectal (colon and rectum) cancers in humans, and also enhances the susceptibility of humans to cigarette smoking eliciting lung and colon cancers.

## 2. Experimental Section 

### 2.1. Population and Group Designation 

The population for this study included 200 cases of primary lung cancer patients and 335 cases of primary colorectal cancer patients recruited from October 2012 to July 2014 at Tangshan People (Tumor) Hospital and Tangshan Worker Hospital. Inclusion criteria: All patients were histopathologically diagnosed as suffering from primary cancers in the lung, composed of small cell cancer (69 cases), squamous cell cancer (56 cases), adenocacinoma (73 cases), and large cell carcinoma (2 cases), as well as in the colon, i.e., colon cancer (130 cases) and in the rectum, i.e., rectal cancer (205 cases), both combined together called colorectal cancer. Notably, colon cancer and rectum cancer share certain similar biological and pathological properties. However, there are many differences between these two cancers, such as the location, function damage, treatment, and prognosis. Furthermore, colon cancer exhibits some different gene mutation patterns in comparison to rectum cancer [30,31,32]. Thus, in this study, we not only carried out a colorectal cancer combined study, but also performed stratified analysis studying these two cancers, respectively. Before collecting blood samples, patients did not undergo any anti–cancer radiation and chemotherapies. No special restriction criteria for patient gender, age, and pathological stage of cancers were required. In the same period, sex-, age-, and resident location-matched 352 healthy individuals undergoing regular physical examination in the same hospitals were selected as controls of the lung cancer and the colorectal cancer groups. Exclusion criteria for control groups: Neither patient relatives nor individuals with familial history of cancer syndromes were allowed to participate in this study. The genotype distribution for the gene polymorphism in the control group must be in conformity with the Hard–Weinberg Equilibrium (HWE) [33]. All participants were Han ethnicity. Using the formulas [34], i.e., $n=2\bar{p}\bar{q}{\left(z_{\alpha }+z_{\beta }\right)}^{2}/{\left(p_{1}-p_{0}\right)}^{2}$ in which $\bar{p}=0.5\times (p_{1}+p_{0})$, $\bar{q}=1-\bar{p}$, *p*_1_ = *p*_0_*RR*/[1 + *p*_0_(*RR* − 1)], *RR* = 2.0, *p*_0_ = 0.4, *α* = 0. 05 (two-sided), *β* = 0.2; the estimated sample size (*n*) needed was at least 121 cases in the cancer groups. Thus, the population size in this study was enough and ideal for comparison between groups. Participants voluntarily joined this study by signature of the medical informed consent. The blood samples (2 mL each) were collected from participants by peripheral venipuncture.

### 2.2. Collection of Epidemiological Data 

Epidemiological data including name, gender, age, ethnicity, address, smoking history, etc., were collected from either patient medical history records or direct communication with patients. For the control group, these data were obtained by the questionnaire survey or directly visiting individuals. Since smoking is a critical environmental risk factor for human lung and colorectal carcinogenesis [27,28,29], our particular interest includes investigating effects of cigarette smoke on the relationship between LOX gene SNP and lung /colorectal carcinogenesis. To exactly obtain the quantitative smoking status in an effort to accurately elucidate smoking effects on the relationship between the LOX G473A SNP and carcinogenesis, only active, rather than passive, smokers were included in the smoking groups. An accumulative smoking volume (ASV), i.e., pack-years (1 pack-year = 1 pack of cigarettes per day for 1 year) [35,36], was used to evaluate a personal smoking status. As known, a cigarette pack contains 20 cigarettes. The average ASVs in this study were 30 pack-years (1 pack of cigarettes per day for 30 years) and 20 pack-years (1 pack of cigarettes per day for 20 years) for the smoking population in the lung cancer and colorectal cancer groups, respectively. Thus, there were 3 smoking status in the tested population: (1) never; (2) mild (<average ASV); and (3) heavy (≥average ASV). Quantification of pack-years smoked is important in clinical diagnosis, where degree of tobacco exposure is correlated to onset of disease, such as lung cancer [37]. In the United States, people who have a 30 pack-year history of smoking are between the ages of 55 and 80, and if they continue to smoke, or have quit in the past 15 years, they are recommended by the Centers for Disease Control and Prevention (CDC) for computed tomography (CT) lung cancer screening [35]. Studies using these criteria for early diagnosis could reduce the mortality rate from lung cancer by 20 percent if people meeting these criteria undergo screening [38]. In addition, more than 85% smokers in cancer groups continuously used cigarettes until diseases were diagnosed, while approximately 70% smokers in control groups in this study currently still use cigarettes. 

### 2.3. Screening of LOX G473A Polymorphism

**Figure 1:**
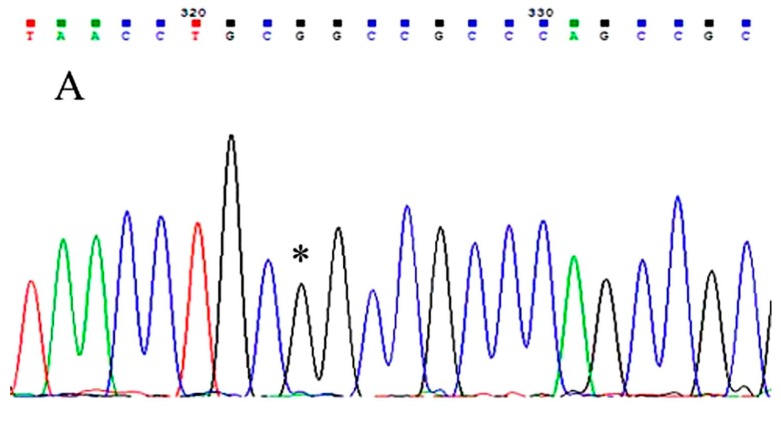

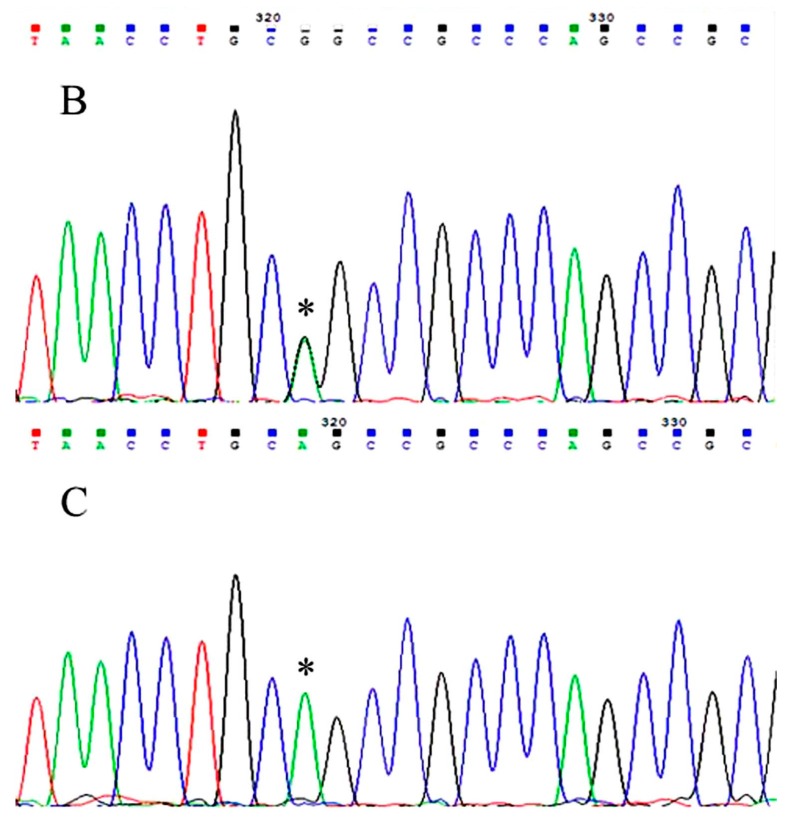
Gene sequence assay indicating LOX G473A polymorphism. GG, the homozygous GG genotype; GA, the heterozygous GA genotype; AA, the homozygous AA genotype. Three genotypes: GG, a black wave in (**A**); GA, a short black wave overlaid with a shorter green wave in (**B**); and AA, a green wave in (**C**) are marked by *.

**Figure 2:**
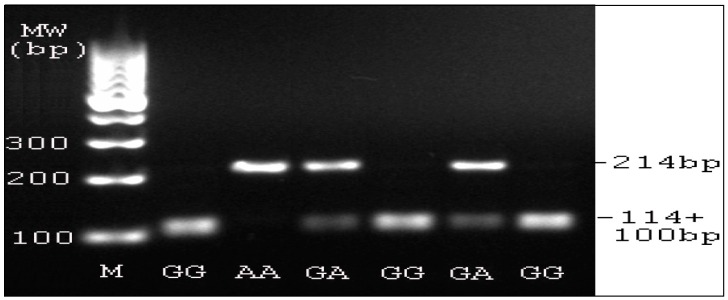
PCR- restriction fragment length polymorphism (PCR-RFLP) analysis for LOX G473A. Total DNA was extracted from the blood sample followed by PCR-amplification using the primers as shown in the text. The PCR product with 214 bp was treated with a specific restriction endonuclease *Not* I with the CG site targeting and analyzed on a 2% agarose gel, stained with ethidium bromide for visualization under ultraviolet light. After electrophoresis, homozygous G alleles which contain the CG site were recognized by DNA bands at 100 and 114 bp in length. An uncut fragment of 214 bp indicated the homozygous A alleles free of the CG site and the heterozygous GA genotype was displayed as a combination of 214, 100, and 114 bp bands. Due to 114 bp and 100 bp DNA being close in the MW, they were smeared as one band in the gel electrophoresis.

Gene sequencing and polymerase chain reaction (PCR)-restriction fragment length polymorphism (PCR-RFLP) assays were used to assess LOX G473A polymorphism. Genomic DNA was extracted from peripheral blood cells using a commercially available Blood genomic DNA miniprep kit according to the manufacturer’s instructions (ZomanBio, Beijing, China). Amplicons containing the LOX G473A fragment were obtained by PCR with primers: forward (F)-5’-TTCCAAGCTGGCTACTCGAC-3’, reverse (R)-5’-CAGGTCTGGGCCTTTCATAA-3’. The PCRs were performed in a total volume of 25 μL reaction mixture containing 0.1 μg genomic DNA (1 μL), F and R primers each 5 μM (1 μL), 50 mM MgCl_2_ 0.8 μL, Platinum^®^ Taq DNA polymerase (Fermentas, Waltham, MA, USA) 0.2 μL, ddH_2_O 18 μL, 10 × PCR buffer 2.5 μL, Reactions were conducted using the thermal cycler (Life Tech., Shanghai, China) under the following conditions: an initial incubation 94 °C for 5 min, denature 94 °C for 30 s, annealing 57 °C for 40 s, extension 72 °C for 30 s, 35 cycles, the final extension 72 °C for 5 min, amplified DNA samples stored at 4 °C. The efficiency of the PCR was confirmed by gel electrophoresis on a 1.5% agarose gel. The PCR amplicons were subjected to DNA sequencing using an ABI 3730XL DNA analyzer (Applied Biosystems, Life Tech. Shanghai, China). The sequence data generated was received as colored electropherograms. (Figure 1). Furthermore, PCR-RFLP analysis was performed as described [15] to identify LOX G473A genotypes. The PCR products were digested 4 h at 37 °C with 10U of the specific restriction endonuclease *Not* I (Life Tech., Shanghai, China), which cut the CG site. The digestion products were then resolved and separated on a 2% agarose gel, stained with ethidium bromide for visualization under ultraviolet light (Figure 2). After electrophoresis, homozygous G alleles which contain the CG site were represented by DNA bands at 100 and 114 bp in length. An uncut fragment of 214 bp indicated the homozygous A alleles and the heterozygous GA genotype was displayed as a combination of 214, 100, and 114 bp bands. 

### 2.4. Statistical Analysis 

The SPSS statistical software package version 16.0 (SPSS Inc., Chicago, IL, USA) was used for statistical analysis. Age comparison between groups was finished by the independent sample *T* test. Gender and smoking status stratified analyses were completed by the chi-square (*χ*^2^) test. Polymorphism results were assessed in control groups for deviation from the Hardy-Weinberg Equilibrium (HWE) test [33] to compare the observed and expected genotype frequencies using the chi-square goodness of fit test. SNP genotype and allele frequencies of LOX were compared between groups by using the chi-square test. Odds ratio (*OR*) and 95% confidence intervals (*CIs*) were calculated according to unconditional logistic regression. The value of α is 0.05, and statistic tests were two-sided. Less than 0.05 of the *p* value was considered to be significant statistically. 

## 3. Results and Discussion

### 3.1. Evaluation of General Characteristics of Study Population

Two hundred lung cancer patients, 335 colorectal cancer patients and 352 healthy controls voluntarily participated in this study. Their age average values were 58.30 ± 9.30, 59.04 ± 10.75, and 58.55 ± 10.29 years old, respectively, for the lung cancer, colorectal cancer, and control groups. The male proportions in the lung cancer, colorectal cancer, and control population groups were 66.00%, 60.00%, and 66.19% while the female proportions in these populations were 34.00%, 40.00%, and 33.83%, respectively (Table 1). Furthermore, the colorectal cancer group was further divided into the colon subgroup with 130 cases and the rectum cancer subgroup with 205 cases. The colon cancer patients with 60.50 ± 9.66 years average age were composed of 56.92% male and 43.08% female, whereas the rectal cancer patients with 58.11 ± 11.32 years average age were composed of 61.95% male and 38.05% female (Table 1). The T and chi-square tests indicated that there were no differences for age and sex distributions between cancer groups and controls (*p* > 0.05) (Table 1). Thus, the cancer samples were matched with the control population at age and sex distribution. 

To assess the genotype frequency status of LOX G473A in the control population, the HWE test was carried out as described [33]. As shown (Table 2), in a total of 352 control peoples the real LOX G473A genotypes such as homozygous GG, heterozygous GA, and homozygous AA were detected in 213, 123, and 16 cases, respectively, whereas these genotypes were expected in 214.09, 120.88, and 17.06 cases, respectively, calculated from the HWE. Deviations of actual samples from Hardy-Weinberg expectations were only −1.09, 2.12, and −1.06, respectively. The chi-square goodness of fit test with one degree of freedom indicated that the real and expected values were not significantly different (*x*^2^ = 0.11, *p* = 0.95), Thus, the control population was in the HWE, which means that the analyzed LOX G473A polymorphic site in the control was not under selection and mutation. As indicated in methods, the minor estimated sample size in this study was 121 cases. Thus, in view of samples in each group, the current study is well able to meet such essential and basic criteria as age and sex matching, enough sample size, good control, etc., for epidemiological studies.

Tobacco use is a leading cause of cancers and of death from cancers [28,29,39]. The lung, colon and rectum are critical targeting organs for cigarette smoke. As noted, the harmful components of smoking not only primarily cause injury to the lung by inhalation, but also damage to the colon-rectum through mixing with saliva and the bloodstream [27,29]. In an effort to identify the relationship between smoke and cancers in the lung, colon, and rectum, a smoke-stratified analysis was performed in this study. As shown in Table 3, in the lung cancer groups with a total of 200 cases, 41% of persons were smokers (note: smokers described in this paper are all active smokers), of which 33% were heavy smokers. In comparison to the control, only 27.7% and 7.67% of the total 352 population were smokers and heavy smokers, respectively. Thus, there were significant differences for smokers and heavy smokers existing between the lung cancer group and the control (both *p* values < 0.01). Although, there were no significant differences for smoker distribution, in colorectal combined, colon, and rectum cancer groups with the controls, marked differences in heavy smoker distribution were found in these groups in comparison to the control (*p* values = 0.01, 0.04, and 0.04 for colorectal, colon, and rectum cancers *vs* control, respectively). Thus, these results suggest that smoking may be correlated with lung and colorectal carcinogenesis.

Cigarette smoke (CS) accounts for 90% of male and 79% of female lung cancers in Western countries [40,41,42]. Notably, Chinese epidemiological studies showed that the attributable risk (AR) of lung cancer associated with smoking was 56.7% for males and 25.5% for females [43]. Such an AR for lung cancer coupled with smoking in China was apparently much lower than those reported in Western countries. In this study, the association of lung cancer with active smoking occurred in 41% of lung cancer cases (Table 3), consistent with the Chinese epidemiological report [43]. Approximately 5 million people in the world die prematurely each year as a result of tobacco use and tobacco smoke exposure [40,41,42]. As reported, nonsmokers exposed to secondhand smoke are called passive smokers, having a 20%–30% chance of developing lung cancer. Occupational exposure, air pollution, radon, etc., are responsible for approximately 20%–30% of lung cancer cases [44]. Tangshan is known as an industrial city in China with heavy industry pollution. These factors may collectively contribute to a higher rate of lung cancer in the nonsmoker population as shown in this study. As a critical cancer risk factor for colon and rectum [29], CS doubles the onset of colorectal adenomas. The adjusted (relative) risk of colorectal cancer (CRC) increases 25% after 10 pack-years of smoking and peaks at approximately 40% after 30 pack-years (1 pack-year = 1 pack of cigarettes per day for 1 year). There is a linear increase in the risk of colorectal cancer with smoking consumption, which is considered to be responsible for 12% of CRC cases [36]. Marked differences in heavy smoker distribution for colorectal, colon and rectum cancers vs. control groups as shown above are consistent with published data.

### 3.2. LOX G473A Polymorphism in Association with Risk for Lung, Colorectal Combined, Colon, and Rectum Cancers 

To assess the relationship between LOX G473A polymorphism and the risk for lung, colorectal combined, colon, and rectum cancers, we performed both genotypic and allelic counts separately and a cross-comparison statistical analysis using the chi-square (*χ*^2^) test. As shown in Table 4, the frequencies of three genotypes of the LOX G473A, i.e., GG, GA, and AA in the control were 60.51%, 34.94%, and 4.55%, respectively, of the tested population. Correspondingly, the frequencies of the G and A alleles of such SNP were 77.98% and 22.02%, respectively, of the total control subjects (Table 5). More impressively, the distribution of LOX G473A variant, either the genotype AA (Table 4) or the allele A (Table 5), was significantly elevated in cancer patients in comparison to the control. The frequencies of LOX 473AA genotype amounted to 17.00%, 10.45%, 10.00%, and 10.79% whereas the frequencies of LOX 473A allele were enhanced to 29.00%, 29.85%, 30.77%, and 29.27% of total patient populations for lung, colorectal combination, colon, and rectum cancers, respectively. *OR* values calculated suggested that LOX 473AA genotype carriers may have a high risks for lung, colorectal, colon, and rectum cancers amounting to 3.84-, 2.74-, 2.75-, and 2.74-fold of those for the LOX473 GG carriers. Furthermore, in comparison to LOX 473G allele individuals, carriers with 473A allele, including GA and AA genotypes, exhibited an increased susceptibility to cancers with the *OR* values of 1.45, 1.50, 1.57, and 1.47, for lung, colorectal, colon, and rectum cancers, respectively. Thus, LOX G473A polymorphism is closely associated with lung, colorectal combined, colon, and rectum carcinogenesis consistent with data published by others [14,16]. It should be noted that this report has also involved some extended studies, for example, lung cancer cases collected included both small cell and non-small cell lung cancer patients as well as a stratified analysis for colon and rectum cancers. 

### 3.3. LOX 473AA-Positive Females Displaying a Higher Susceptibility to Lung, Colorectal Combined, Colon, and Rectum Cancers 

To compare the sex difference in LOX G473A genotype frequency distribution in the tested population, we completed the unconditional logistic regression test with *p* values adjusted for age and smoking status. The sex difference in LOX G473A genotype frequency in patients and controls is tabulated in Table 6. As an example in the lung cancer group (Table 6, A. Lung Cancer group), 12.5% male and 4.5% female population carried the LOX 473AA genotype, whereas in the control group, only 3.69% male and 0.85% females displayed this LOX gene variant. In comparison to the same sex population with LOX 473GG, lung cancer risk was increased to 3.23-fold (*OR* value = 3.23, 95%*CI* = 1.53–6.82) and 5.25-fold (*OR* value = 5.25, 95%*CI* = 1.33–20.80) for male and female populations with LOX 473AA genotype, respectively (both *p* value ≤ 0.05). Furthermore, B. Colorectal Cancer group in Table 6 showed that LOX 473AA-positive men and women both displayed a higher potential for suffering from colorectal cancer, reaching 1.51 (*OR* value = 1.51, 95%*CI* = 1.04–2.20) and 2.29-fold (*OR* value =2.29, 95%*CI* = 1.20–4.38) (both *p* value ≤ 0.05), respectively, of those in the same sex population with the genotype of LOX 473GG. Notably, the LOX 473GA-positive women whose LOX gene contained only one A allele also exhibited a high susceptibility to colorectal cancer in comparison to the LOX 473GG female population (*OR* value = 1.90, 95%*CI* = 1.11–3.26). For colon cancer (Table 6, C. Colon Cancer group), only the LOX 473AA positive female population was shown to have a high risk (*OR* = 2.27, 95%*CI =* 1.10–4.70, *p* ≤ 0.05), while there was no difference between LOX 473AA male populations and their controls. LOX A allele-carrying female populations including GA and AA genotypes were strongly susceptible to rectum cancer, such that the risks were increased to 1.91-fold for the LOX 473GA population (*OR* = 1.91, 95%*CI =* 1.03–3.54) and 2.25-fold for the LOX 473AA population, (*OR* = 2.25, 95%*CI =* 1.11–4.53) (both *p* value ≤ 0.05), respectively, in comparison to the population with the LOX 473GG control. Although the LOX473AA male population also had a high risk for rectum cancer, their *OR* value (*OR* = 1.53, 95%*CI =* 1.01–2.32) was apparently lower than that in the LOX 473GA or AA female population (Table 6, D. Rectum Cancer group). These results suggest that there were different susceptibilities to colon and rectum cancers for individuals carrying different LOX G473A variants. Briefly, using LOX 473GG as control (*OR* = 1), the OR values for female vs. male were 5.25 vs. 3.23, 2.29 vs. 1.51, 2.27 vs. 1.45, and 2.25 vs. 1.53, for lung, colon-rectal combined, colon, and rectum cancers, respectively. Thus, in comparison to the male population, the LOX 473AA female population was coupled with a higher susceptibility to lung, colorectal, colon, and rectum cancers.

In consistency with our findings, incidences for breast cancer [17], cervical cancer [18], and ovarian cancer [19], i.e., major cancers for woman, were also significantly increased in the LOX G473A variant female population. Thus, LOX G473A polymorphism may be implicated in modulation of estrogen-directed mechanisms (see below) for promotion of cancer development in woman. This hypothesis should be further demonstrated in the future. 

### 3.4. Smoke Targeting LOX473AA-Positive Individuals in Association with Higher Risks for Cancers in the Lung and Colon 

Cigarette smoke is a common risk factor critical for lung and colorectal carcinogenesis [27,28,29]. Smoking or heavy smoking has been indicated relevant to lung and colorectal cancer development (Table 3). LOX G473A variant-positive populations have also been shown having a higher susceptibility to lung and colorectal cancer onset (Table 4). Here, we further evaluated the modulating effects of smoking on the lung and colorectal carcinogenesis in the LOX G473A variant-carrying population by performing unconditional logistic regression analysis. As shown in Table 7, A. Lung Cancer group, the smoking population who were LOX 473AA-positive displayed a higher susceptibility to lung cancer, reaching 3.25-fold of the corresponding LOX 473 GG controls, indicating the collective impact of dual factors, i.e., the LOX G473A polymorphism and smoking, on carcinogenesis. Table 7 also showed that neither colorectal combined cancer risk (Table 7, B. Colorectal Cancer group) nor rectum cancer risk (Table 7, D. Rectum Cancer group) was enhanced in the LOX 473AA-carrying smoking population in comparison to those in the LOX 473GG controls. In contrast, the LOX 473AA-positive population with smoking exhibited an increased risk for colon cancer, amounting to 2.11-fold of that in the LOX 473GG-positive control, while no statistical significance in the colon cancer risk was found between the LOX 473AA- and 473GG-carrying non-smoking population (Table 7, C. Colon Cancer group). It should be noted that the LOX 473AA-positive nonsmoking population also exhibited high risks for lung cancer (Table 7, A. Lung Cancer non-smoking group) and colorectal combined cancer (Table 7, B. Colorectal Cancer non-smoking group), indicating the LOX G473A polymorphism as a unique factor affecting carcinogenesis. Furthermore, we also analyzed the relationship of LOX G473A genotype frequency distribution in cancer and control populations with the various smoking degrees. As shown in Table 8, A., the Lung Cancer group contained 82 smokers (41% of total cases), including 66 heavy smokers (33% of total cases) and 16 mild smokers (8% of total cases). Among heavy smokers, 16 cases were LOX 473AA-positive in the cancer group; in contrast, no one case was LOX 473AA-positive in the control population (*p* = 0.007). Thus, heavy smokers with the LOX 473AA genotype may be more sensitive than those with the LOX 473GG genotype to lung carcinogenesis. Consistent with the result as described above (Table 7, C. Colon Cancer group), heavy smokers with LOX 473AA displayed a higher risk for colon cancer in comparison to those with LOX 473 GG (*OR* = 18.20, 95%*CI =* 1.47–225.19) (Table 8, C. Colon Cancer heavy smoker group). These results indicated that LOX G473A variant-positive individuals are a critical population for cigarette smoke targeting inducing cancers in the lung and colon. Different modulation effects of cigarette smoke on the relationship of the LOX G473A polymorphism with colon and rectum cancers may reflect different gene mutation patterns between these two organs [30,31,32].

### 3.5. LOX and Cancers 

LOX was considered as a tumor suppressor beginning with the finding that cloned mouse *ras* recession gene (*rrg*) cDNA in sequence was nearly identical with the rat LOX cDNA (>96%) [2]. Expression of transfected LOX cDNA suppressed Ha-*ras*-induced cell transformation [2] and altered chromatin packing in the nuclei [45] indicating a *ras*-suppressor effect of LOX. Furthermore, normal rat kidney fibroblasts expressing LOX antisense became transformed displaying anchorage-independent growth and upregulating p21-*ras* [46]. LOX mRNA was downregulated in a variety of spontaneous human cancers [47] such as bronchogenic carcinoma [48], gastric cancers [49], head and neck squamous cell carcinoma [50], etc. Somatic mutation of the LOX gene reduced expression of this enzyme associated with human colorectal tumors [51]. Basic fibroblast growth factor (bFGF) is a substrate of LOX. Oxidation of bFGF by LOX blocked the proliferation of bFGF-stimulated cells and bFGF-autocrine transformed cells with highly tumorigenic potential [4]. Interestingly, LOX and its oxidized substrates exist within the nuclei of cultured vascular smooth muscle cells (VSMC) and 3T3 fibroblasts [7]. Histone H1 and H2, critical nuclear structural proteins, have been identified as a substrate of LOX [8,9]. Repression of *BCL*2, a proto-oncogene, by proLOX and LOX propeptide inhibited transformed the phenotype of lung cancer cells [52]. Thus, modulating the expression of oncogenes such as *ras* and *BCL2*, regulating the activity of growth factors such as bFGF, and stabilizing nuclear proteins such as histone H1 and H2 and thus maintaining chromatin and nuclear stabilities, may be key mechanisms for LOX tumor suppression. 

On the other hand, high levels of LOX were detected in the hypoxia-stage of some tumors, facilitating metastasis [3,53]. LOX has chemotactic activity [5,6]. As an extracellular signal, LOX stimulates stress fiber formation and focal adhesion assembly [6], enhancing cell migration, which implicates in mechanisms for tumor metastasis in hypoxia conditions. Solid tumors are often associated with deficiency of blood supply in the central areas of the tumor, thus inducing hypoxia. LOX is a hypoxia-responsive gene since its gene promoter contains the hypoxia response element (HRE) [54,55]. The activation of the LOX gene initiates upregulation of its substrates, such as collagen and elastin [1], providing a media for cell movement. In addition, hypoxia can also initiate the epithelial–mesenchymal transition (EMT) for cancer cells to gain migratory and invasive properties [56,57]. Inhibition of LOX by BAPN blocked the process of EMT [58]. Thus, hypoxia-elicited EMT in association with remodeling of the ECM collectively facilitated metastasis of cancer cells [3,53]. In review of LOX’s dual roles in carcinogenesis, any genetic and epigenetic modulation of LOX expression including SNP should damage its tumor suppressor functions and alter cancer cell behaviors. 

### 3.6. LOX G473A Polymorphism and Cancers 

The LOX gene contains 7 exons encoding the full length of the LOX protein [12]. LOX is initially synthesized as a 46 kDa preproenzyme. Following signal peptide cleavage and N-glycosylation, the resulting 50 kDa proenzyme is secreted and then proteolysed to the 18 kDa propeptide and the 32 kDa functional species in the ECM [1]. The LOX G473A (rs1800449) is located at the exon1 [12,13]. Mutation of LOX gene 473G to 473A results in a change of Arg at residue 158 to Gln (LOX Arg158Gln) in the LOX propeptide [13]. Since it was discovered [13], the LOX G473A polymorphism has been studied as a focus for its relationship with carcinogenesis [14,15,16,17,18,19,20,21]. The LOX G473A polymorphism is often positively detectable in the Eastern population, such as the Chinese and Korean [14,15,16,17,18,19,20,21]. In this study, the GA and AA genotype frequencies were 34.94% and 4.55% of the total tested control population (*N* = 352), respectively (Table 4). Notably, in cancer groups, these frequencies of the LOX G473A polymorphism were significantly increased. For example, the LOX 473AA positive frequencies were elevated to 17.00%, 10.45%, 10.00%, and 10.79% of total tested cases (lung cancer *N* = 200, colorectal cancer *N* = 335, colon cancer *N* = 130, rectum cancer *N* = 205) in lung, colon-rectum, colon, and rectum cancer patients, respectively (Table 4). Our study has shown that in comparison to LOX473GG genotype carriers, individuals with LOX473AA exhibited a higher susceptibility to lung, colon-rectum, colon, and rectum cancers, respectively. LOX propeptide has been shown to display tumor suppressor activity [52,59,60]. The LOX G473A variant resulting in an Arg158Gln substitution in the LOX propeptide may impair its ability for tumor inhibition, a potential mechanism for LOX G473A polymorphism correlation with carcinogenesis. 

### 3.7. Cigarette Smoke and Cancers

Cigarette smoke (CS), a chemical mixture, contains more than 4800 different compounds including oxidants, heavy metals, and carcinogens, that individually or collectively initiate or promote carcinogenesis in the lung [28,61,62]. Furthermore, CS compounds target the colon and rectum via mixing with saliva and the bloodstream, eliciting the onset of cancers in these organs [36]. The carcinogenic chemical compounds of tobacco smoke, such as acetaldehyd, benz-pyrenes, aromatic amines, and N-nitrosamines, form DNA adducts, binding to DNA molecules and disrupting normal gene function and replication [36,44]. Interestingly, recent studies have indicated the role of female hormones such as estrogen in cigarette smoke carcinogenesis in the lung and breast via targeting the endocrine system [63,64,65], Cadmium, a major carcinogen in cigarette smoke, functions as a metalloestrogen, promoting breast and lung cancer onset [66]. Downregulation of LOX by cadmium and cigarette smoke condensate [67] may interfere with the LOX tumor suppressor function *via* activation of the estrogen pathway. This may explain a higher susceptibility to lung, colorectal, colon, and rectum cancers as well as breast and ovarian cancers, etc. for woman with LOX G473A variants, a highly frequent LOX gene abnormality. Our study has identified that smoking significantly increased the risk in the LOX 473AA-positive population of developing lung and colon cancers (Table 7). A further study into degree of smoking has also shown that the LOX G473A polymorphism markedly enhanced susceptibilities to lung and colon cancers in a heavy-smoking population (Table 8). Different responses of colon and rectum organs in LOX473AA carriers to cigarette smoke suggest that smoking carcinogens elicit cancers in these two organs, possibly by activation of different mechanisms. Thus, our results have demonstrated that LOX G473A polymorphism apparently elevated human sensitivity to environmental carcinogens such as cigarette smoking in different organs with various degrees. 

## 4. Conclusions 

This study investigated the frequency of the LOX gene G473A SNP in North Chinese population and identified its relationship with carcinogenesis in the lung, colon-rectum combined, colon, and rectum. Results indicated that the LOX G473A variant-positive population displayed an enhanced susceptibility to lung, colorectal, colon, and rectum cancers. The risk for cancer onset in these organs is higher in the LOX 473AA genotype-carrying females than males. The LOX G473A polymorphism apparently elevated human sensitivity to cigarette smoking carcinogens for eliciting cancers in the lung and colon. Thus, the LOX G473A polymorphism positively correlates with carcinogenesis. These findings suggest that the LOX G473A polymorphism may be used as an ideal intrinsic biomarker for prediction or diagnosis of carcinogenesis in human. 

## Figures and Tables

**Table 1:** Age and sex distribution in tested population.

Characteristics		Population Number (%)		*χ*^2^	*p*
		Cancer Group	Control Group		
**A. Lung Cancer **		200 (100.00)	352 (100.00)		
Age ($\bar{x}\pm s$)		58.30 ± 9.30	58.55 ± 10.29	−0.35 *^t^*	0.73
Sex	Male *N* (%)	132 (66.00)	233 (66.19)		
	Female *N* (%)	68 (34.00)	119 (33.81)	0.00	0.96
**B. Colorectal Cancer **		335 (100.00)	352 (100.00)		
Age ($\bar{x}\pm s$)		59.04 ± 10.75	58.55 ± 10.29	0.62 *^t^*	0.54
Sex	Male *N* (%)	201 (60.00)	233 (66.19)		
	Female *N* (%)	134 (40.00)	119 (33.81)	2.83	0.09
**C. Colon Cancer **		130 (100.000)	352 (100.00)		
Age ($\bar{x}\pm s$)		60.50 ± 9.66	58.55 ± 10.29	1.88 *^t^*	0.61
Sex	Male *N* (%)	74 (56.92)	233 (66.19)		
	Female *N* (%)	56 (43.08)	119 (33.81)	3.53	0.06
**D. Rectum Cancer **		205 (100.00)	352 (100.00)		
Age ($\bar{x}\pm s$)		58.11 ± 11.32	58.55 ± 10.29	0.46 *^t^*	0.64
Sex	Male *N* (%)	127 (61.95)	233 (66.19)		
	Female *N* (%)	78 (38.05)	119 (33.81)	1.02	0.31

Age difference analysis performed by the *T* test, *^t^* represents *T* value; sex difference analysis performed by the *χ*^2^ test. *N*, population number.

**Table 2:** LOX G473A genotype frequency analysis for control group by Hardy-Weinberg Equilibrium (HWE) test.

Genotypes	Genotype Frequencies (Total *N* = 352)		Deviation	*χ*^2^	*p*
	Real *N*	Expected *N*			
GG	213	214.06	−1.06		
GA	123	120.88	2.12	0.11	0.95
AA	16	17.06	−1.06		

HWE performed by the chi-square (*χ*^2^) goodness of fit test. *N*, population number. GG, the homozygous GG genotype; GA, the heterozygous GA genotype; AA, the homozygous AA genotype.

**Table 3:** Smoker distribution in tested population.

Smoking Status	Population *N* (%)		*χ*^2^	*p*
	Cancer Groups	Control Groups		
**A. Lung Cancer **	200 (100.00)	352 (100.00)		
Non-smoking	118 (59.00)	256 (72.73)		
Smoking	82 (41.00)	96 (27.27)	11.00	<0.01 *
Mild	16 (8.00)	69 (19.60)		
Heavy	66 (33.00)	27 (7.67)	48.00	<0.01 *
**B. Colorectal Cancer **	335 (100.00)	352 (100.00)		
Non-smoking	262 (78.21)	256 (72.73)		
Smoking	73 (21.79)	96 (27.27)	2.78	0.10
Mild	25 (7.46)	52 (14.77)		
Heavy	48 (14.33)	44 (12.50)	6.63	0.01 *
**C. Colon Cancer **	130 (100.00)	352 (100.00)		
Non-smoking	100 (76.92)	256 (72.73)		
Smoking	30 (23.08)	96 (27.27)	0.87	0.35
Mild	10 (7.69)	52 (14.77)		
Heavy	20 (15.38)	44 (12.50)	3.97	0.04 *
**D. Rectum Cancer **	205 (100.00)	352 (100.00)		
Non-smoking	162 (79.02)	256 (72.73)		
Smoking	43 (20.98)	96 (27.27)	2.74	0.10
Mild	15 (7.32)	52 (14.77)		
Heavy	28 (13.66)	44 (12.50)	4.42	0.04 *

Smoker distribution analysis performed by the *χ*^2^ test. Mild smoker, a person who used cigarettes <average ASV, i.e., 30 pack-years in the lung cancer group and 20 pack-years in the colorectal cancer groups; heavy smoker, a person who used cigarettes ≥average ASV. * indicates significance statistically. Those *p* values lower than 0.01 such as 0.008, 0.005, 0.003, etc. all were expressed as <0.01, other *p* values equal or larger than 0.01 are shown by the exact numbers in this or following Tables.

**Table 4:** LOX G473A genotype frequency distribution in tested population.

Genotypes	Genotype Frequencies (%)		*χ*^2^	*p*	*OR* (95%*CI*)
	Cancer Groups	Control Groups			
**A. Lung Cancer**	200 (100.00)	352 (100.00)			
GG	118 (59.00)	213 (60.51)	-	-	1.00 (Ref.)
GA	48 (24.00)	123 (34.94)	2.93	0.09	0.70 (0.47–1.05)
AA	34 (17.00)	16 (4.55)	19.00	<0.01	3.84 (2.03–7.24) *
**B. Colorectal Cancer**	335 (100.00)	352 (100.00)			
GG	170 (50.75)	213 (60.51)	-	-	1.00 (Ref.)
GA	130 (38.80)	123 (34.94)	2.99	0.08	1.32 (0.96–1.82)
AA	35 (10.45)	16 (4.55)	10.61	<0.01	2.74 (1.47–5.12) *
**C. Colon Cancer**	130 (100.00)	352 (100.00)			
GG	63 (48.46)	213 (60.51)	-	-	1.00 (Ref.)
GA	54 (41.54)	123 (34.94)	3.32	0.07	1.48 (0.97–2.27)
AA	13 (10.00)	16 (4.55)	6.79	0.01	2.75 (1.25–6.02) *
**D. Rectum Cancer**	205 (100.00)	352 (100.00)			
GG	107 (52.20)	213 (60.51)	-	-	1.00 (Ref.)
GA	76 (37.01)	123 (34.94)	1.22	0.27	1.23 (0.85–1.78)
AA	22 (10.79)	16 (4.55)	8.82	<0.01	2.74 (1.38–5.43) *

LOX G473A genotype frequency analysis performed by the chi-square (*χ*^2^) test; *OR*, odd ratio; *CI*, confidence interval. Ref., using GG as reference 1; * indicating significance statistically.

**Table 5:** LOX G473A allele frequency distribution in tested population.

Allele	Allele Frequencies (%)		*χ*^2^	*p*	*OR* (95%*CI*)
	Cancer Groups	Control Groups			
**A. Lung Cancer**	400 (100.00)	704 (100.00)			
G	284 (71.00)	549 (77.98)	-	-	1.00 (Ref.)
A	116 (29.00)	155 (22.02)	6.72	0.01	1.45 (1.09–1.91) *
**B. Colorectal Cancer**	670 (100.00)	704 (100.00)			
G	470 (70.15)	549 (77.98)	-	-	1.00 (Ref.)
A	200 (29.85)	155 (22.02)	10.99	0.01	1.5 0 (1.18–1.92) *
**C. Colon Cancer**	260 (100.00)	704 (100.00)			
G	180 (69.23)	549 (77.98)	-	-	1.00 (Ref.)
A	80 (30.77)	155 (22.02)	7.89	0.01	1.57 (1.15–2.16) *
**D. Rectum Cancer**	410 (100.00)	704 (100.00)			
G	290 (70.73)	549 (77.98)	-	-	1.00 (Ref.)
A	120 (29.27)	155 (22.02)	7.33	0.01	1.47 (1.10–1.93) *

LOX G473A allele frequency analysis performed by the chi-square (*χ^2^*) test; G, the G allele; A, the A allele; *OR*, odd ratio; *CI*, confidence interval; Ref., using G as reference 1. Total allele frequency number = total genotype frequency number × 2; total number for each allele = homozygous number × 2 + heterozygous number; * indicating significance statistically.

**Table 6:** Analysis for sex difference in LOX G473A genotype frequency distribution.

Genotypes	Genotype Frequencies (%)		*p*	*OR* (95%*CI*)
	Cancer Groups	Control Groups		
**A. Lung Cancer**	200 (100.00)	352 (100.00)		
**Male**	132 (66.00)	233 (66.19)		
GG	72 (36.00)	133 (37.78)	-	1.00 (Ref.)
GA	35 (17.50)	87 (24.72)	0.26	0.75 (0.46–1.24)
AA	25 (12.50)	13 (3.69)	<0.01	3.23 (1.53–6.82) *
**Female**	68 (34.00)	119 (33.81)		
GG	46 (23.00)	80 (22.73)	-	1.00 (Ref.)
GA	13 (6.50)	36 (10.23)	0.19	0.60 (0.29–1.27)
AA	9 (4.50)	3 (0.85)	0.02	5.25 (1.33–20.80) *
**B. Colorectal Cancer**	335 (100.00)	352 (100.00)		
**Male**	201 (60.00)	233 (66.19)		
GG	105 (31.34)	133 (37.78)	-	1.00 (Ref.)
GA	75 (22.39)	87 (24.72)	0.67	1.09 (0.73–1.63)
AA	21 (6.27)	13 (3.69)	0.03	1.51 (1.04–2.20) *
**Female**	134 (40.00)	119 (33.81)		
GG	65 (19.40)	80 (22.73)	-	1.00 (Ref.)
GA	55 (16.42)	36 (10.23)	0.02	1.90 (1.11–3.26) *
AA	14 (4.18)	3 (0.85)	0.01	2.29 (1.20–4.38) *
**C. Colon Cancer**	130 (100.00)	352 (100.00)		
**Male**	74 (56.92)	233 (66.19)		
GG	36 (27.69)	133 (37.78)	-	1.00 (Ref.)
GA	31 (23.85)	87 (24.72)	0.47	1.23 (0.71–2.13)
AA	7 (5.38)	13 (3.69)	0.14	1.46 (0.88–2.43)
**Female**	56 (43.08)	119 (33.81)		
GG	27 (20.77)	80 (22.73)	-	1.00 (Ref.)
GA	23 (17.69)	36 (10.23)	0.09	1.83 (0.91–3.69)
AA	6 (4.62)	3 (0.85)	0.03	2.27 (1.10–4.70) *
**D. Rectum Cancer **	205 (100.00)	352 (100.00)		
**Male**	127 (61.95)	233 (66.19)		
GG	69 (33.66)	133 (37.78)	-	1.00 (Ref.)
GA	44 (21.46)	87 (24.72)	0.92	0.98 (0.61–1.56)
AA	14 (6.83)	13 (3.69)	0.04	1.53 (1.01–2.32) *
**Female**	78 (38.05)	119 (33.81)		
GG	38 (18.54)	80 (22.73)	-	1.00 (Ref.)
GA	32 (15.61)	36 (10.23)	0.04	1.91 (1.03–3.54) *
AA	8 (3.90)	3 (0.85)	0.02	2.25 (1.11–4.53) *

Analysis for sex difference in LOX G473A genotypes performed by the unconditional logistic regression test; *p* values adjusted for age and smoking status; *OR*, odd ratio; *CI*, confidence interval. Ref., using GG as reference 1; * indicating significance statistically.

**Table 7:** Analysis for smoking status difference (non-smoking and smoking) in LOX G473A genotype frequency distribution.

Genotypes	Genotype Frequencies (%)		*p*	*OR* (95%*CI*)
	Cancer Groups	Control Groups		
**A. Lung Cancer**	200 (100.00)	352 (100.00)		
Non-smoking	118 (59.00)	256 (72.73)		
GG	75 (37.50)	156 (44.30)	-	1.00 (Ref.)
GA	26 (13.00)	90 (25.60)	0.08	0.62 (0.37–1.05)
AA	17 (8.50)	10 (2.84)	<0.01	3.97 (1.70–9.29) *
Smoking	82 (41.00)	96 (27.27)		
GG	43 (21.50)	57 (16.19)	-	1.00 (Ref.)
GA	22 (11.00)	33 (9.38)	0.67	0.86 (0.43–1.73)
AA	17 (8.50)	6 (1.70)	0.03	3.25 (1.15–9.16) *
**B. Colorectal Cancer **	335 (100.00)	352 (100.00)		
Non-smoking	262 (78.21)	256 (72.73)		
GG	136 (40.60)	156 (44.30)	-	1.00 (Ref.)
GA	98 (29.25)	90 (25.60)	0.23	1.25 (0.87–1.81)
AA	28 (8.36)	10 (2.84)	<0.01	1.77 (1.21–2.59) *
Smoking	73 (21.79)	96 (27.27)		
GG	34 (10.15)	57 (16.19)	-	1.00 (Ref.)
GA	32 (9.55)	33 (9.38)	0.14	1.67 (0.85–3.27)
AA	7 (2.09)	6 (1.70)	0.41	1.30 (0.70–2.43)
**C. Colon Cancer**	130 (100.00)	352 (100.00)		
Non-smoking	100 (76.920	256 (72.73)		
GG	54 (41.54)	156 (44.30)	-	1.00 (Ref.)
GA	38 (29.23)	90 (25.60)	0.38	1.25 (0.76–2.05)
AA	8 (6.15)	10 (2.84)	0.10	1.51 (0.92–2.48)
Smoking	30 (23.08)	96 (27.27)		
GG	9 (6.92)	57 (16.19)	-	1.00 (Ref.)
GA	16 (12.31)	33 (9.38)	0.07	2.33 (0.94–5.75)
AA	5 (3.85)	6 (1.70)	0.04	2.11 (1.02–4.38) *
**D. Rectum Cancer**	205 (100.00)	352 (100.00)		
Non-smoking	162 (79.02)	256 (72.73)		
GG	82 (40.00)	156 (44.30)	-	1.00 (Ref.)
GA	60 (29.26)	90 (25.60)	0.26	1.28 (0.84–1.96)
AA	20 (9.76)	10 (2.84)	<0.01	1.86 (1.24–2.80) *
Smoking	43 (20.98)	96 (27.27)		
GG	25 (12.20)	57 (16.19)	-	1.00 (Ref.)
GA	16 (7.81)	33 (9.38)	0.71	1.16 (0.53–2.55)
AA	2 (0.98)	6 (1.70)	0.71	0.84 (0.34–2.09)

Analysis for smoking status difference in LOX G473A genotypes performed by the unconditional logistic regression test; *p* values adjusted for age and sex; *OR*, odd ratio; *CI*, confidence interval. Ref., using GG as reference 1; * indicates significance statistically.

**Table 8:** Analysis for smoking status difference (heavy and mild smoking) in LOX G473A genotype frequency distribution.

Genotypes	Genotype Frequencies (%)		*p*	*OR (*95%*CI)*
	Cancer Groups	Control Groups		
**A. Lung Cancer**	200 (100.00)	352 (100.00)		
Smoking (Total)	82 (41.00)	96 (27.27)		
Heavy-smoking	66 (33.00)	27 (7.67)		
GG	32 (16.00)	17 (4.83)	-	1.00 (Ref.)
GA	18 (9.00)	10 (2.84)	0.47	0.67 (0.25–1.90)
AA	16 (8.00)	0 (0.00)	<0.01	- * ^f^
Mild-Smoking	16 (8.00)	69 (19.60)		
GG	11 (5.50)	40 (11.36)	-	1.00 (Ref.)
GA	4 (2.00)	23 (6.53)	0.55	0.68 (0.19–2.41)
AA	1 (0.50)	6 (1.70)	0.63	0.76 (0.25–2.34)
**B. Colorectal Cancer **	335 (100.00)	352 (100.00)		
Smoking (Total)	73 (21.79)	96 (27.27)		
Heavy-smoking	48 (14.33)	44 (12.50)		
GG	22 (6.57)	27 (7.67)	-	1.00 (Ref.)
GA	22 (6.57)	16 (4.55)	0.21	1.77 (0.73–4.27)
AA	4 (1.19)	1 (0.28)	0.16	2.36 (0.72–7.77)
Mild-Smoking	25 (7.46)	52 (14.77)		
GG	12 (3.58)	30 (8.52)	-	1.00 (Ref.)
GA	10 (2.99)	17 (4.83)	0.40	1.57 (0.54–4.57)
AA	3 (0.90)	5 (1.42)	0.98	1.01 (0.44–2.33)
**C. Colon Cancer**	130 (100.00)	352 (100.00)		
Smoking (Total)	30 (23.08)	96 (27.27)		
Heavy-smoking	20 (15.38)	44 (12.50)		
GG	6 (4.62)	27 (7.67)	-	1.00 (Ref.)
GA	10 (7.69)	16 (4.55)	0.18	2.31 (0.68–7.84)
AA	4 (3.08)	1 (0.28)	0.02	18.20 (1.47–225.19) *
Mild-Smoking	10 (7.69)	52 (14.77)		
GG	3 (2.31)	30 (8.52)	-	1.00 (Ref.)
GA	6 (4.62)	17 (4.83)	0.71	0.64 (0.06–6.87)
AA	1 (0.77)	5 (1.42)	0.73	1.54 (0.13–18.76)
**D. Rectum Cancer**	205 (100.00)	352 (100.00)		
Smoking (Total)	43 (20.98)	96 (27.27)		
Heavy-smoking	28 (13.66)	44 (12.50)		
GG	16 (7.80)	27 (7.67)	-	1.00 (Ref.)
GA	12 (5.85)	16 (4.55)	0.56	1.35 (0.50–3.65)
AA	0 (0.00)	1 (0.28)	-	-
Mild-Smoking	15 (7.32)	52 (14.77)		
GG	9 (4.39)	30 (8.52)	-	1.00 (Ref.)
GA	4 (1.95)	17 (4.83)	0.76	0.80 (0.21–3.15)
AA	2 (0.98)	5 (1.42)	0.91	0.90 (0.13–6.00)

Analysis for smoking status difference in LOX G473A genotypes performed by the unconditional logistic regression test; *p* values adjusted for age and sex; *OR*, odd ratio; *CI*, confidence interval; Mild smoker, a person used cigarette < average ASV, i.e., 30 pack-years in the lung cancer group and 20 pack-years in the colorectal cancer groups; heavy smoker, a person used cigarette ≥ average ASV. Ref., using GG as reference 1; * indicates significance statistically. * ^f^ Since 0 case for the genotype AA in the control group, the difference for cancer and control groups was analyzed by the Fisher exact test, the actual *p* value = 0.007.
